# Attitudes and factors influencing organ donation decision-making in Damascus, Syria: a cross-sectional study

**DOI:** 10.1038/s41598-023-45388-6

**Published:** 2023-10-24

**Authors:** Jameel Soqia, Jamal Ataya, Rawan Alhomsi, Horiya Soqia, Ameer Kakaje, Rakan Saadoun, Ammar Hamzeh

**Affiliations:** 1 Faculty of Medicine, Damascus University, Damascus, Syria; 2 German-Syrian Research Society e.V., Frankfurt, Germany; 3 Faculty of Medicine, University of Aleppo, Aleppo, Syria; 4 Department of Ophthalmology, Faculty of Medicine, Al-Baath University, Homs, Syria; 5 Faculty of Dentistry, Damascus University, Damascus, Syria; 6 University Hospital Geelong, Barwon Health, Geelong, Victoria Australia; 7 Medical Faculty Mannheim, Ruprecht Karls University Heidelberg, Heidelberg, Germany; 8 Department of Otorhinolaryngology-Head and Neck Surgery, Mannheim Medical Center, Mannheim, Germany; 9 Department of Plastic Surgery, The University of Pittsburgh, Pittsburgh, USA

**Keywords:** Health care, Health policy, Medical ethics, Public health

## Abstract

Organ donation is vital to saving lives, but its success depends on people's willingness to donate organs. This descriptive cross-sectional survey aimed to investigate attitudes towards organ donation in Damascus, Syria. Understanding attitudes is crucial for the success of organ donation programs, especially in countries with similar settings. This study was a descriptive cross-sectional survey aimed at understanding patients’ attitudes towards organ donation in Damascus, Syria. Data was collected through a carefully constructed validated survey through face-to-face interviews. 600 participants were randomly interviewed, 62.8% agreed to donate their organs after death, with helping others being the primary reason. Religious beliefs were the primary reason for organ refusal in males, while for females, it was lack of knowledge and religious beliefs. However, there were no significant differences between genders or educational level and age groups in the acceptance of organ donation. The percentage of those who agree to donate their organs after death encourages taking an effective step to build an integrated donation system, not just a center. Bearing in mind that there is no correlation with age, gender or even educational level, which means that the system may include different groups of society.

## Introduction

Organ donation is a critical aspect of modern medicine that has the potential to save many lives. However, the success of organ donation programs is dependent on people's willingness to donate their organs after death^1,2^. In the United States of America (USA), the need for organ transplants is dire, with more than 120,048 men, women, and children on the waiting list for life-saving procedures. Tragically, every single day, 21 individuals lose their lives while awaiting organ transplants^2^.

Despite the importance of organ donation, there is a significant variation in the acceptance rates for organ donation across different countries and regions, with figures ranging from 31.3 to 85%^2–6^. Several factors have been identified as influencing people’s attitudes towards organ donation, such as religious beliefs, cultural norms, knowledge, awareness, and trust in the health system^7^. Therefore, understanding the specific context and characteristics of each population is essential for designing effective strategies to promote organ donation.

In Syria, a previous study conducted in Aleppo found that a significant majority of participants, around 51%, expressed willingness to donate their organs^8^. However, this study was limited by its small sample size and its focus on one city in the north of Syria. Therefore, there is a gap in the literature regarding the attitudes towards organ donation in Syria, especially in the capital of Syria, Damascus. Regrettably, Syria lacks a formalized donation system or a structured donor card program, thereby rendering organ procurement predominantly reliant upon individual contributions, often stemming from the illicit organ commercialism^8^. Consequently, the scope of organ transplantation procedures transcends the confines of specific healthcare institutions.

To address this gap, this study aimed to investigate patients’ attitudes towards organ donation in Damascus, Syria, by conducting a descriptive cross-sectional survey. The survey aimed to collect information on demographic characteristics and attitudes towards organ donation among patients attending outpatient clinics at three public hospitals. The specific objectives of this study were:

- To assess the level of willingness to donate organs among patients in Damascus.
- To identify the factors associated with willingness to donate organs among patients in Damascus.
- To explore the reasons for willingness or unwillingness to donate organs among patients in Damascus.

The importance of this study lies in its contribution to our understanding of patients’ attitudes towards organ donation in Syria, and provides valuable insights into the factors influencing willingness to donate organs. With a significant proportion of the population in need of life-saving organ transplants, understanding attitudes towards donation is crucial to the success of organ donation programs. The results of this study have the potential to inform policies aimed at improving organ donation programs in Syria and other countries with similar settings.

## Methods

### Study design

In order to better understand patients' attitudes on organ donation, we performed a descriptive cross-sectional survey. Patients receiving medical care at public hospitals in Damascus, Syria were the focus of this research. We used a cross-sectional approach, which entailed the collecting of data at a particular moment in time, because the success of organ donation programs strongly depends on people's willingness to donate their organs. Data were collected by face-to-face interviews with participants who were chosen at random from the halls and waiting rooms of several hospital departments. The patients were approached and asked to participate in the study. The survey was carried out between November 6 and December 7, 2022, for a total of one month. This time frame was selected to guarantee thorough data collection and a representative sampling of the population. Also, the length of time allows for the collecting of enough data to guarantee consideration of the broader population in Syria.

### Participants and data collection

We applied a strict methodology to carry out this investigation. The criteria for participation in the research were as follows: the participants had to be patients or their companions et al.-Mouwasat Hospital, a public hospital that is the largest and most advanced medical center in Syria; they had to be 18 years of age or older; and they had to agree to participate in the study and sign an informed consent form. The research was conducted in a public hospital that is the largest and most advanced medical center in Syria (Al-Mouwasat Hospita). The hospital is affiliated with the University of Damascus and serves as an academic center for teaching and research. The hospital receives patients from all over the country, not only from Damascus, as it provides specialized and tertiary care services that are not available in other regions. Therefore, the population interviewed in this study can be considered representative of the broader population in Syria, as it reflects the diversity and complexity of the Syrian society.

The study eliminated any missing data, and individuals who were suffering from dangerous condition and could not perform an face-to-face interview. The data were carefully examined and proofread after entering to remove any potential biases that could have been introduced during face-to-face interviews. The aims of the study were clearly explained to the participants, and they were also given assurances about the privacy of their answers. Both patients and their companions voluntarily participated. The study was carried out in complete conformity with the Helsinki Declaration, and it was approved by the Ethics Committee of the Faculty of Medicine at the University of Damascus (ID:1769, 17/10/2022).

### Questionnaire

The survey that was used in the study was carefully constructed to collect detailed and reliable information about the attitudes and opinions that the respondents had toward organ donation. The survey underwent a thorough procedure to confirm its accuracy in meaning and ideas, and it was adapted from other previously validated questionnaires^8^. The questionnaire was also evaluated by experts in the field of organ donation and transplantation, who provided feedback on its content and structure. The questionnaire was then pilot-tested with 68 participants from the same population as the main study, and the results were analyzed using descriptive statistics and Cronbach’s alpha test. The sample size for the pilot study was determined by using a formula that was applied in previous studies^9^. The formula considered a significance level of 0.05 and a confidence level of 95%, which resulted in a required sample size of 59 participants for the pilot study. Cronbach's Alpha test was used to assess the survey's reliability, and it produced an exceptional internal consistency score of 0.855. Results showed that the survey was an extremely trustworthy tool for acquiring information about participant opinions regarding organ donation. A clear and organized method for gathering data was supplied by the bulk of the survey's questions, which were presented in a closed style.

There were two main sections of the research questionnaire (6 questions). The initial part (3 questions) of this investigation was to collect participants' demographics data, such as their gender, age, and level of education. The second Section (3 questions) focused on gathering information related to participants' attitudes towards organ donation. This section aimed to evaluate their willingness to donate their organs, the reasons behind their decision, and their understanding of the donation process. The questions were closed-ended.

### Ethical approval and consent to participate

The authors assert that all procedures contributing to this work comply with the ethical standards of the relevant national and institutional committees on human experimentation and with the Helsinki Declaration of 1975, as revised in 2008. The Ethical Committee approved this study in the Faculty of Medicine at Damascus University, Syria (ID: 1769, 17/10/2022). All our methods were carried out following relevant guidelines and regulations. Informed consent was obtained from all the participants included in the study. We explained the purpose of the study to each participant, and it was all voluntary. No names were taken, so we provided anonymous data collection.

### Statistical analysis

The data collected through the electronic questionnaire on Google forms was exported to Excel for analysis. Statistical analysis was carried out using SPSS Inc. version 23 software package. Chi-square test was used to identify any correlation between the demographic variables and the attitude towards organ donation. A *p*-value of less than 0.05 was considered statistically significant.

### Sample size

To determine the appropriate sample size (n), the research team utilized Cochran's Sample Size Formula. The calculations were based on several factors, including a 95% confidence level (represented by Z = 1.96), a margin of error of 5% (represented by e), and an estimated proportion (p) of the population that possesses the attribute of interest of 50% (or 0.5). The value of q was calculated as 1 − p:

$$n=\frac{{Z}^{2}pq}{{e}^{2}}$$

The required Sample size (n) for this study, applying the previous formula, is 385.

## Results

### Demographic characteristics

600 participants (N = 600) were randomly interviewed, 44.5% (n = 267) of participants were between 30 and 49 years old, 32% (n = 192) participants were between 50 and 70 years old. Additionally, 72.3% of the sample were females (n = 434), and 27.7% were males (n = 166). 46.3% (n = 278) had only a primary school education. Other details and characteristics are shown in Table 1.

**Table 1:** Sample characteristics.

Variables		Frequency (n)	Percent (%)
Gender	Male	166	27.7
	Female	434	72.3
Age groups	18–29 years	119	19.8
	30–49 years	267	44.5
	50–70 years	192	32
	Above 70 years old	21	3.5
Educational level	Unschooled	87	14.5
	Primary school education	278	46.3
	Secondary school education	105	17.5
	University or Institution	130	21.7

### Attitudes towards organs donation and its associated factors

**Figure 1:**
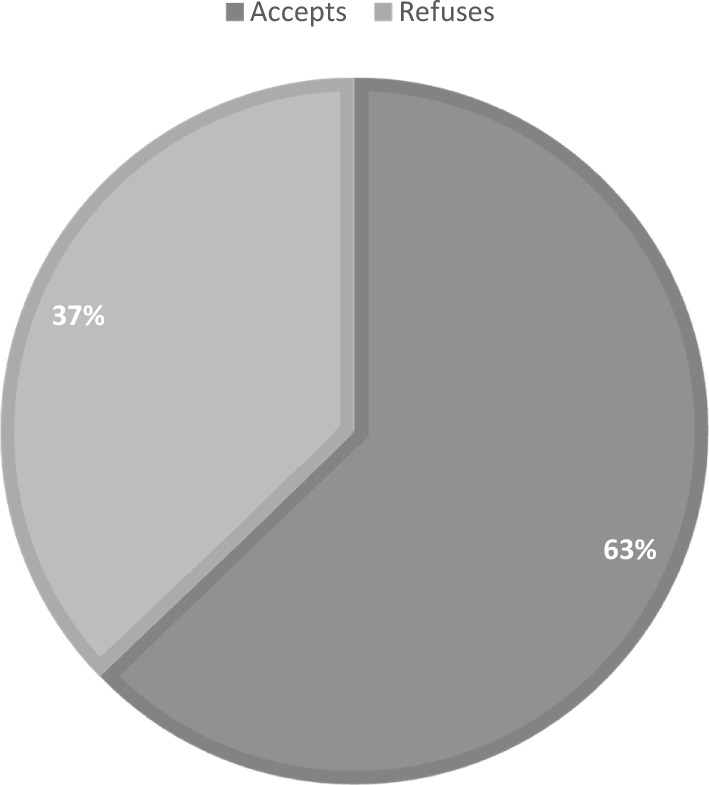
Organ donation acceptance rates after death among participants.

**Figure 2:**
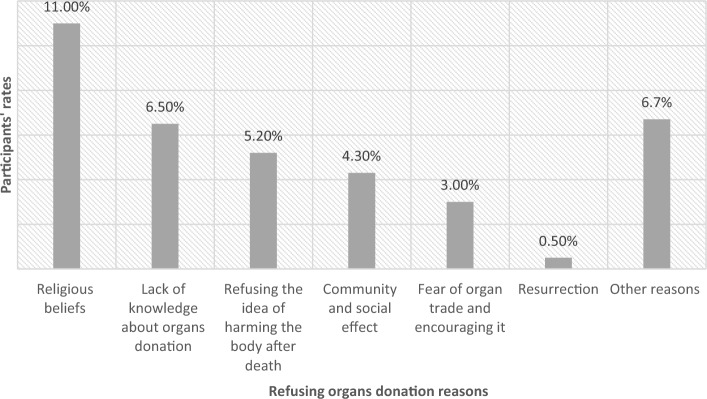
Refusing organs donation reasons among participants.

In the study, it was found that out of the total 600 participants, 62.8% (n = 377) agreed to donate their organs after their death (Fig. 1). On the other hand, 37.2% (n = 223) refused organ donation. The reasons behind refusal were religious beliefs (11%), lack of knowledge about organ donation (6.5%), unwillingness to cause harm to the body after death (5.2%), social and community effects (4.3%), fear of organ trade and its promotion (3%), belief in Resurrection (0.5%), and other reasons (6.7%) (Fig. 2).

**Figure 3:**
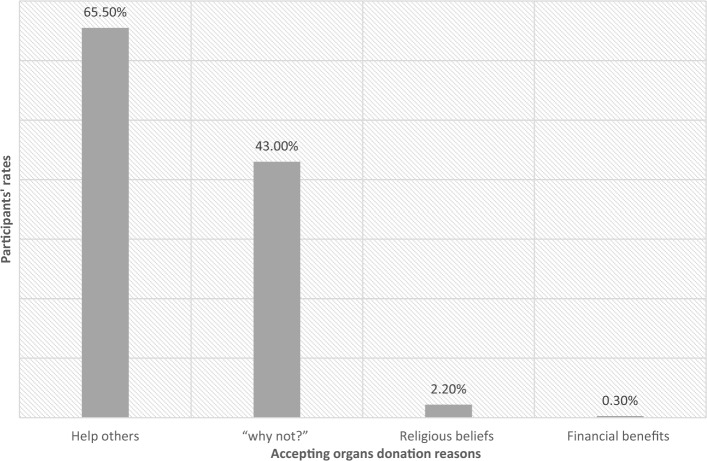
Accepting organs donation reasons among participants.

In contrast, the reasons behind accepting organ donation were primarily based on the intention to help others (65.5%), followed by a nonchalant attitude of "why not?" (43%), religious beliefs (2.2%), and financial benefits (0.3%) (Fig. 3). Note that participants were allowed to select multiple choices here.

Further analysis of the data showed that for males who refused to donate their organs, religious beliefs were the primary reason (n = 34). In contrast, for females, the leading causes of refusal were religious beliefs and lack of knowledge about organ donation (n = 32 for each).

The study did not find any significant differences between females and males in their willingness to donate their organs after death. Statistical analysis showed no significant variation, X^2^ (1, N = 600) = 0.389, *p* = 0.533. Similarly, there were no significant differences observed in the acceptance of organ donation based on educational level (*p* = 0.382) and age groups (*p* = 0.059).

## Discussion

Organ donation is the key to achieving self-sufficiency in addressing organ failure cases in Syria. While kidney transplantation is prevalent in Syria^10^, other tissue transplants are scarce, with only one kidney transplant in 1979, three heart transplants in 1990, and two liver transplants in 2017 and 2019^8,11^. In 2019 alone, 5407 renal transplants were performed^12^, and now, kidney, cornea, and bone marrow transplants are possible^13,14^. Despite legal provisions prohibiting organ commercialism^15,16^, most kidney transplants in Syria rely on it^10^, which poses a risk to society. The only solution is to promote tissue donation through the healthcare system. Therefore, the present study aims to evaluate people’s willingness to donate their body parts through a questionnaire administered to those present at a central hospital in the capital. Academic intellectuals have also expressed their opinions on organ donation, which may influence the public perception and policy making on this issue. A systematic review and a study highlighted the complexity and multifactorial nature of organ donation decision-making, and suggested modifying educational programs, social behavior and school curricula to promote organ donation^7^. Some studies focused on the opinions of university students, as they represent a potential source of donors and future health professionals, and found that knowledge, awareness, attitude, willingness and trust are important factors that affect their opinions on organ donation^3,8,17^.

The results of our study demonstrate that 62.8% of respondents were willing to donate their organs after death, which is higher than the rates reported in prior research conducted in Syria, Saudi Arabia, South Korea, and Morocco (51%, 42%, 60.9%, and 57.6%, respectively)^8,18–21^, but it is slightly lower than the rate reported in a Jordanian study (72%)^22^.

Our study found that, similarly to prior research conducted in Syria, Turkey, and Saudi Arabia^8,23,24^, the primary motivation for organ donation was to help others. This was particularly evident during the survey period, which coincided with a devastating earthquake in Syria and Turkey that brought people together in solidarity, especially from the healthcare perspective, where exceptional care is needed, and organ transplantation plays a crucial role. Another significant motivation was the notion of “why not.” Given that we are discussing donation after death, this suggests that these individuals are not opposed to the principle of donation and are not hindered by any religious or legal barriers. Rather, they prioritize their own safety and recognize that the body holds no importance after death, making organ donation an easy decision.

In terms of obstacles to organ donation, religious beliefs are the most significant deterrent, as observed in previous studies^19,25,26^. Syria has a predominantly Muslim population, though both Islam and Christianity do not prohibit organ donation. People are cautious about whether donation is a religious taboo or not. It was not confirmed for some time whether organ donation was halal or haram in Islam, but a fatwa was issued by Dar Al Iftaa in Saudi Arabia in 1995, allowing donation. In Syria, the supreme Islamic religious authorities issued a decision in 2001 allowing organ procurement from deceased persons, provided a relative's approval is obtained^12^. Nevertheless, some individuals may not be aware of these legislative developments, leading to a lack of knowledge about their religion's stance on organ donation after death^27^. However, a previous study in Saudi Arabia reported the opposite, with religious beliefs serving as an incentive for organ donation^18^. The second most significant obstacle is a lack of knowledge about organ donation, which is unsurprising given the weak scientific awareness and media coverage in this area. Religious beliefs are equally obstructive to both genders, although women are more affected by the lack of knowledge. We speculate that men are more knowledgeable because they are more involved in society, as they are primarily responsible for providing for their families in our Eastern cultures.

There is no effect of age on the decision to donate. In contrast to studies that showed that approval of organ donation is lower among the elderly^20,28^. We did not find any significant difference among different age groups. We have never witnessed any large-scale awareness campaigns at any time affecting any of the age groups, and the idea of organ donation as a whole is new to the entire Syrian society, young or old. Nor was gender associated with consent to donate, similar to a study^23^. In contrast to Jordanian and German studies where females are more willing to donate^28–30^. In addition, there is no association between the educational level and the decision to donate, as the issue of organ donation has never been part of any educational path. However, in Moroccan and German studies, a positive effect of a higher level of education was found^20,28^.

In addition to the challenge of a shortage of donors, which our survey results suggest we may overcome, there are other obstacles to organ transplantation in Syria. We require a donation system and a medical team specializing in organ transplantation at the national level. This includes the need for medical teams to perform transplants, train healthcare staff, and prepare hospitals for successful transplantation^16^. While the Syrian government initiated a project to start liver transplantation, it was interrupted by the war, and even bone marrow transplants were not adequately organized due to the conflict^16^. There are two models for the donation system: opt-in and opt-out. However, morally and legally defensible policies for opt-out are challenging in many developing countries^31^. Furthermore, although we have an organ donation center, it is currently ineffective^11,32^. It is critical to revitalize the center and establish an institution that safeguards donors from fraud or organ trade. All aspects of the donation process should be transparent and publicly available^33^, so that people can trust doctors and the healthcare system as a whole. Lack of trust in doctors has been identified as a significant barrier to organ donation in previous studies^26^. It is crucial to increase awareness of organ donation through social media, healthcare professionals, and educational institutions. Religious awareness may also contribute to the dissemination of organ donation^34,35^, so it should be an integral part of organ donation awareness campaigns. The family plays a significant role in overturning the desire of a deceased relative to donate their organs^36^ and can influence the donation decision positively or negatively. Therefore, it is essential to create an awareness platform that targets families as a whole, rather than just individuals, to ensure better awareness and a confirmed decision. By promoting organ donation among prominent individuals, such as notables and medical professionals, Turkey successfully bolstered its organ donation rates, demonstrating the effectiveness of enlisting influential social figures in advocating for this cause^37^.

### Implications and recommendations

The findings of this paper have important implications for improving the organ donation situation in Syria and other similar contexts. First, it is essential to raise public awareness about the religious and legal aspects of organ donation, as well as the benefits and risks involved. This can be done through media campaigns, educational programs, and community outreach initiatives that target both men and women of different age groups and educational levels. Second, it is necessary to establish a national organ donation system that ensures the ethical and transparent allocation of organs, respects the wishes and rights of donors and recipients, and provides adequate support and follow-up for both parties. Third, it is vital to develop the medical infrastructure and human resources required for organ transplantation, such as specialized teams, training programs, and hospital facilities. These steps would help overcome the barriers to organ donation and transplantation in Syria and increase the availability and quality of this life-saving procedure.

### Limitations

The survey questions were limited and did not cover other significant factors that may influence participants' decisions regarding organ donation. We did not explore the topic of organ donation before death. Furthermore, the study was conducted in only one major hospital. Additionally, This study was limited by the use of face-to-face interviews, which may have created a social desirability bias and affected the validity, reliability, and generalizability of the data. While individuals may express support for organ donation to help others, their inclination toward financial gain through organ sales remains uncertain, a predilection that may be accentuated amid the backdrop of Syria’s economic crisis. Discussing organ donation in a society characterized by a pervasive organ trade presents inherent challenges. Consequently, an imperative proposition emerges: the imperative eradication of organ trade as a preliminary measure, followed by the endeavor to disseminate the organ donation within the society. The variance among religions was not part of our discourse, as the focal point of our research was not to scrutinize of religion's direct influence, but rather to identify the fundamental impediments encountered in the context of donation principles. It is noteworthy that future research endeavors should comprehensively explore the influence of religion on this subject matter.

## Conclusion

Based on the results, the percentage of those who agree to donate their organs after death encourages taking an effective step to build an integrated donation system, not just a center. Bearing in mind that there is no correlation with age, gender or even educational level, which means that the system may include different groups of society.

## Data Availability

The datasets generated and analyzed during the current study are not publicly available to protect participants’ privacy but are available from the corresponding author on reasonable request.
